# Micro-computed tomographic evaluation on the quality of single-cone obturation using a modified passive-deflation sealer injection needle: an in vitro study

**DOI:** 10.1186/s12903-024-04232-7

**Published:** 2024-04-20

**Authors:** Zhuo Chen, Nuo Chen, Yanling Yang, Wei Fan

**Affiliations:** State Key Laboratory of Oral & Maxillofacial Reconstruction and Regeneration, Key Laboratory of Oral Biomedicine Ministry of Education, Hubei Key Laboratory of Stomatology, School & Hospital of Stomatology, Wuhan University, Wuhan, People’s Republic of China

**Keywords:** Root canal obturation, Sealer, Void, Single-cone obturation, micro-CT, iRoot SP

## Abstract

**Objectives:**

This study aimed to design a modified passive-deflation sealer injection needle and investigate its ability to improve obturation quality of single-cone technique through assessing the distribution of voids in root canals using micro-computed tomography (micro-CT).

**Materials and methods:**

Forty-eight mandibular incisors were divided into eight groups (*n* = 6), according to the taper of root canal preparation (0.06 or 0.04), the needle used for sealer injection (modified or commercial iRoot SP injection needle), and the obturation method (iRoot SP sealer-only or single-cone obturation). After obturation, each specimen was scanned by micro-CT. The volumetric percentage and distribution of all voids were first analyzed and compared among groups, then the open and closed voids were separately analyzed and compared among single-cone obturation groups.

**Results:**

Compared to commercial needle groups, modified needle groups showed much less voids, especially in the apical root canal part (*P* < 0.05). Besides, the modified needle groups produced much less open voids than commercial needle groups despite the root canal taper (*P* < 0.05).

**Conclusions:**

The modified passive deflation sealer injection needle could effectively improve the quality of single-cone obturation through reducing intra-canal voids, especially open voids throughout the root canal, thus might possibly be developed as an effective intra-canal sealer delivering instrument.

## Background

Root canal obturation is an essential step in root canal treatment, aiming to seal the intra-canal space and prevent bacterial invasion and reinfection [1]. Unsatisfactory root canal obturation normally contains gaps and voids in the filling material, which would serve as pathways for micro-leakage or shelters for infectious bacteria [2]. It is reported that voids among gutta-percha, sealer and canal wall could be a major cause of root canal reinfection [3].

Current root canal obturation techniques include cold lateral compaction, warm vertical compaction, and single-cone technique. The single-cone obturation technique involves the use of a single gutta-percha point matching the prepared root canal together with the root canal sealer. This technique shows many advantages over the cold lateral and warm vertical compaction techniques such as limited root canal enlargement, simplified procedures, and almost no pressure on the root canal wall and thermal damage to the periodontal membrane [4, 5]. Despite this, the single-cone technique seems likely to produce voids in root canal fillings, with a volumetric percentage of voids ranged from 1.6 to 9.7% in different studies [6–10].

Due to the low flowability and incompatibility of gutta-percha on dentin surface, sealer needs to be used to seal the gaps among gutta-percha or between gutta-percha and root canal wall [11]. IRoot SP is a bio-ceramic root canal sealer widely used in single-cone obturation due to its superior sealing property, bio-compatibility, and chemical stability [12, 13] However, when the sealer is injected into the root canal, especially the root canal with mature apex, it would push the air inside the canal towards the apical foramen to form an apical air bubble [14]. This phenomenon is commonly observed in root canal irrigation, which is known as the vapor lock effect [15]. Once formed, these bubbles would be difficult to remove by simply inserting gutta-percha cones, thus forming voids in root canal fillings and posing a negative effect on the root canal obturation [14, 16]. Therefore, preventing the voids formation in the root canal fillings, especially in the apical canal area, would be very important for the obturation quality of single-cone technique. To precisely assess the volume of voids within the root canal fillings, micro-computed tomography (micro-CT) with high resolution is required due to its precision, safety and repeatability in detecting the voids within root canal fillings [17].

Based on the above knowledge, the purpose of this study was to design a modified passive-deflation sealer injection needle, and investigate its ability to improve the quality of single-cone obturation through assessing the distribution of voids in root canals using the micro-CT with high resolution.

## Materials and methods

### Sample selection

Utilizing G*Power 3.1 9.7 (Universitat Dusseldorf, Dusseldorf, Germany), ANOVA (fixed-effects, omnibus, one-way) was employed for calculating the sample size. Based on our previous experimental data [18], α was set at 0.05, effect size at 0.6, and power β at 0.8. The sample size was determined as 6 per group.

With the approval (2022-A20) of the Ethics Committee of School & Hospital of Stomatology, Wuhan University, forty-eight mandibular incisors extracted due to periodontal disease were collected. The inclusion criteria for the teeth used in this study were as follows: single-rooted mandibular incisors with complete root development and similar root lengths, root canal curvature less than 15°, no defect or resorption, and no history of dental treatment. Teeth with immature apices, absorption or fractures, multiple root canals, curvature greater than 15°, caries, or those that had undergone root canal therapy were excluded. The teeth were cleaned using a scalpel to remove soft tissue, calculus and bone tissue, and then preserved in normal saline for following experiments.

### Root canal preparation

All teeth were decoronated using a low-speed saw, preserving 12 mm of each root. A 10# K-file (Dentsply, Ballaigues, Switzerland) was inserted into root canal until the file tip was visible at the apical foramen. The inserting length was measured, and the working length was set as 0.5 mm shorter than the inserting length. The roots were then randomly divided into eight groups (A ∼ H), with 6 specimens in each group. After scouting and negotiation with 10# manual K-files, the root canals were prepared using Orodeka Plex 2.0 (Orodeka, Jining, China) rotary instruments according to manufacture’s instructions. Roots in groups A, B, E and F were prepared to 25# and 0.06 taper, while roots in groups C, D, G and H were prepared to 25# and 0.04 taper. Each file was discarded after 5 uses. During preparation, 5mL of 1% sodium hypochlorite solution was used as the irrigant. The root canals were dried by paper points and the apices were sealed by paraffin. All operations were performed by the same endodontist.

### Design of modified needles

**Fig. 1:**
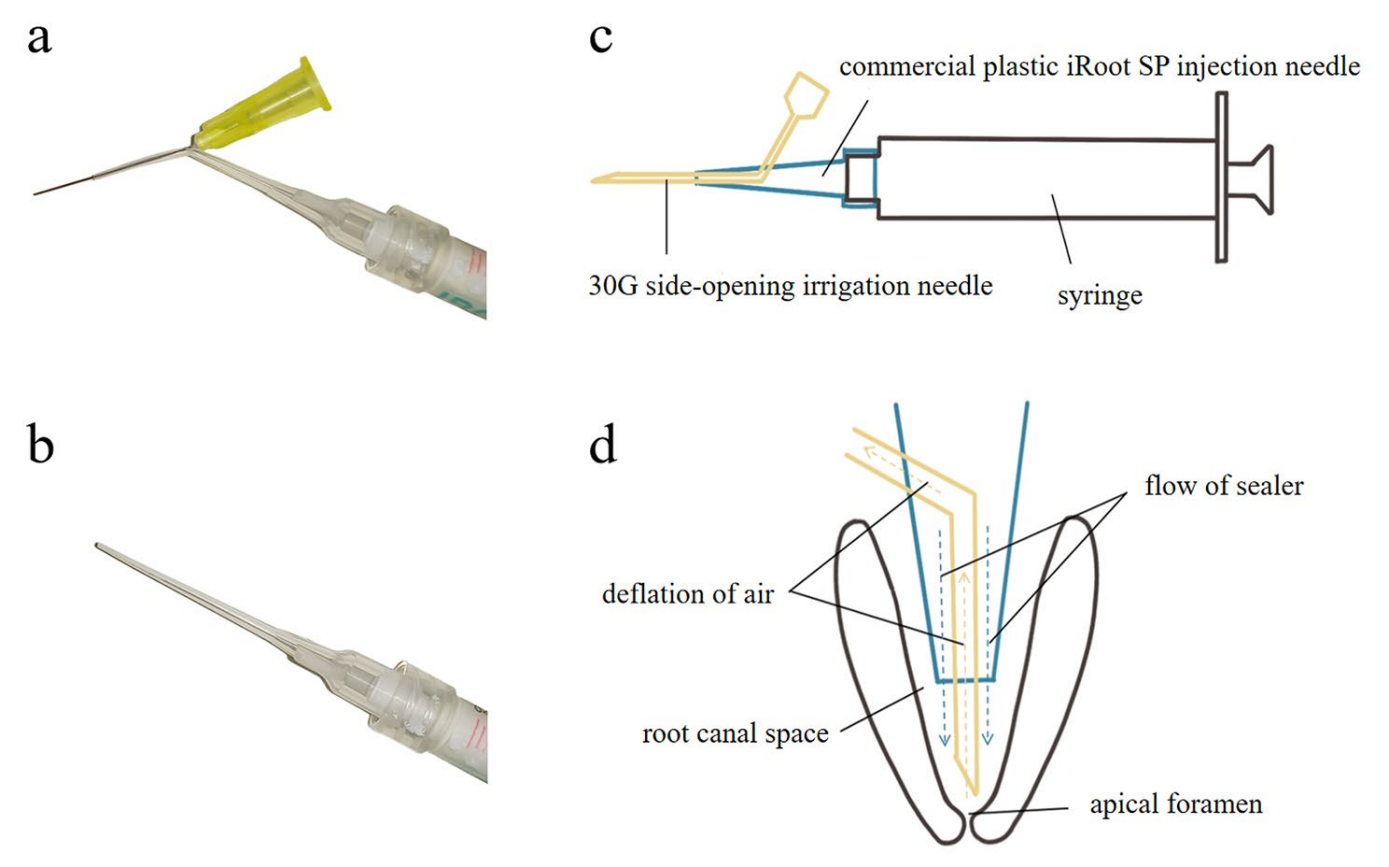
Modified passive-deflation sealer injection needle and its working mechanism. (**a**) Picture of the modified passive-deflation injection needle; (**b**) Picture of the commercial plastic iRoot SP injection needle; (**c**) Illustration of the modified passive-deflation sealer injection needle; (d) Working mechanism of the modified needle

Modified passive-deflation sealer injection needles (Fig. 1a) were made using commercial iRoot SP plastic needles (Innovative Bioceramix, Vancouver, Canada) (Fig. 1b) and 30G side-opening irrigation needles (Renwoo, Shanghai, China). As we had described in our previous study [18], a small hole was made on the side of the commercial needle, and the 30G irrigation needle was inserted into the commercial needle through the hole and extended 5 ∼ 7 mm beyond the commercial needle tip (Fig. 1c). To ensure the irrigation needle tip could reach to 1 ∼ 2 mm from the apex, it was extended 5 mm from the commercial needle tip in 0.06 taper root canals, and 7 mm in 0.04 taper root canals. In this way, during iRoot SP injection, the sealer would enter the root canal from the gap between the commercial needle and the irrigation needle, while the air in the apical area would be passively deflated from the root canal through the irrigation needle (Fig. 1d).

### Root canal obturation

IRoot SP sealer (Innovative Bioceramix, Vancouver, Canada) was injected using commercial needles in groups A, C, E and G. The needle was connected to the iRoot SP syringe and inserted into the root canal to the maximal depth. The sealer was then slowly injected until the root canal was filled with sealer. Then the needle was slowly withdrawn from the root canal with continuous slow injection of the sealer. In groups B, D, F and H, modified needles were used for sealer injection with the same procedure. Each needle was discarded after single use.

For groups E ∼ H, after sealer injection, gutta-percha points with matched sizes were slowly inserted into root canals to the working length. Extra gutta-percha at the canal orifice was severed using the Beefill 2in1® device (VDW, München, Germany). Then the canal orifice was sealed with flowable resin composite. All procedures were performed by the same endodontist.

All specimens were then stored at 37 °C and 100% humidity for one week to allow the sealer to set completely.

### Micro-CT scanning

Specimens were scanned using a SkyScan 1276 high-resolution micro-CT scanner (SkyScan, Kontich, Belgium). The parameters were set at 100 kV, 200µA, spatial resolution of 6 μm, and rotation step of 0.25°. The cross-section images were obtained through SkyScan CT analysis software (SkyScan, Kontich, Belgium). Mimics 20.0 (Materialise, Leuven, Belgium) was used for three-dimensional visualization analysis and the volumetric measurement of voids and filling materials (gutta-percha and sealer) for each specimen. The whole root was divided into 3 equal parts (coronal, middle and apical) with each 4 mm long. The volumetric percentages of voids in the whole root canal and each canal part were calculated separately. The distribution of voids was analyzed and compared among groups. Additionally, in groups E ∼ H, voids were classified as closed voids (confined in the filling material) and open voids (communicating with the root canal wall), and their volumetric percentages in the whole and each part of root canal were further calculated respectively. The volumetric percentage of voids (total, closed or open) = volume of voids (total, closed or open) / (volume of total voids + volume of fillings) * 100%. To avoid observer bias, the above analysis and calculation processes were independently conducted by a single experienced examiner .

### Statistical analysis

Statistical analysis was performed using GraphPad 9.0 (GraphPad Software, Boston, USA) and SPSS 24.0 (IBM Corp, Somers, USA), with all data expressed as mean ± standard deviation (SD). The normality was assessed using the Sharpiro-Wilk test. Due to the non-normal distribution of data from multiple groups, the Kruskal-Wallis test was used to compare the volume of voids in different root canal parts among groups and within each group, and the Mann-Whitney test was applied for mutual comparisons between groups. The significance level was set as *P* < 0.05.

## Results

### Difference of total voids in sealer-only injection (SOI) groups

**Fig. 2:**
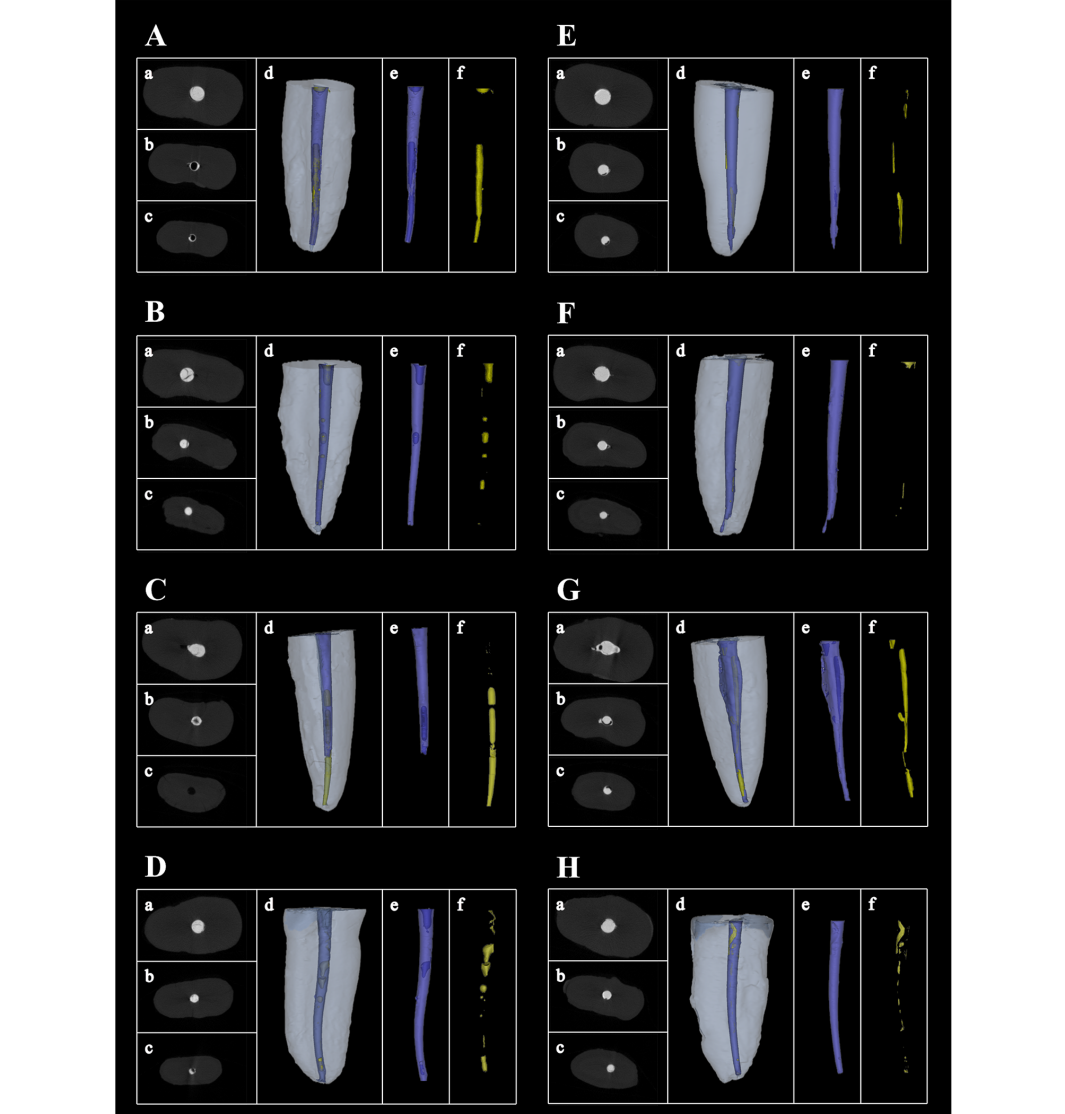
Representative cross-sections and three-dimensional reconstructions of filling material (blue) and voids (yellow) in groups A ∼ H. (**a**) Representative cross-sections of the coronal 1/3; (**b**) Representative cross-sections of the middle 1/3; (**c**) Representative cross-sections of the apical 1/3; (**d**) Representative three-dimensional reconstructions of the whole root; (**e**) Representative three-dimensional reconstructions of filling material; (**f**) Representative three-dimensional reconstructions of voids

The volumetric percentages of voids in each group were summarized in Table 1. The representative cross-sections and three-dimensional reconstructions of voids and fillings were shown in Fig. 2. Voids were identified in all groups. For SOI groups (A ∼ D), the overall volumetric percentages of voids in modified needle groups were significantly lower than those in commercial needle groups despite the canal taper (A vs. B, C vs. D, *P* < 0.01 and < 0.05 respectively). In the coronal 1/3, there were no significant differences among all groups (*P* > 0.05). In the middle 1/3 of 0.06 tapered canal, the volumetric percentage of voids in the modified needle group was significantly lower than that in the commercial needle group (A vs. B, *P* < 0.05), but no significant difference was found in 0.04 tapered canals of this root part. In the apical 1/3, volumetric percentages of voids in modified needle groups were significantly lower than that in commercial needle groups despite the canal taper (A vs. B, C vs. D, *P* < 0.01 and < 0.05 respectively). There were no statistical differences between different taper groups when using the same needle at each canal part (A vs. C, B vs. D, *P >* 0.05).

### Difference of total voids in single-cone obturation (SCO) groups

Similarly, for the overall volumetric percentages of voids in SCO groups (E ∼ H), modified needle groups were significantly lower than commercial needle groups despite the canal taper (E vs. F, G vs. H, *P* < 0.05 and < 0.01 respectively). In the coronal 1/3, modified needle groups also showed significantly lower volumetric percentages of voids than commercial needle groups in both 0.06 and 0.04 tapered canals (E vs. F, G vs. H, *P* < 0.05). In the middle 1/3, a significantly lower volumetric percentage of voids was only detected in the modified needle plus 0.04 rather than 0.06 tapered group (G vs. H, *P <* 0.01). In the apical 1/3, a significantly lower volumetric percentage of voids existed in groups using modified needles in either 0.04 or 0.06 tapered canals (E vs. F, G vs. H, *P* < 0.01). There were no significant differences between different taper groups using the same needle at each canal part (E vs. G, F vs. H, *P* > 0.05).

**Table 1:** Volumetric Percentages of Voids (mean ± SD) in Different Groups

Filling Method	Group	Taper	Needle Type	Volume of Voids (%)				*P* value*
				Overall	Coronal	Middle	Apical	
SOI	A	0.06	commercial	29.68 ± 7.926^a^	11.04 ± 10.230^A^	40.01 ± 18.256^a, B^	72.53 ± 20.797^a, C^	0.002
	B	0.06	modified	10.60 ± 4.760^a^	12.31 ± 4.938^A^	9.50 ± 7.169^a, A^	9.06 ± 9.004^a, A^	0.587
	C	0.04	commercial	25.37 ± 12.852^b^	4.95 ± 5.012^A^	32.01 ± 36.296^B^	76.89 ± 38.317^b, C^	0.049
	D	0.04	modified	9.30 ± 5.132^b^	5.63 ± 6.852^A^	9.66 ± 7.475^A^	18.61 ± 21.374^b, A^	0.296
SCO	E	0.06	commercial	2.04 ± 0.761^c^	1.61 ± 0.596^c, A^	1.71 ± 2.116^A^	4.96 ± 2.163^c, B^	0.019
	F	0.06	modified	0.26 ± 0.302^c^	0.20 ± 0.311^c, A^	0.30 ± 0.685^A^	0.43 ± 0.557^c, A^	0.822
	G	0.04	commercial	4.86 ± 3.015^d^	7.84 ± 8.358^d, A^	4.86 ± 3.920^d, A^	6.53 ± 4.399^d, A^	0.810
	H	0.04	modified	0.46 ± 0.767^d^	0.81 ± 1.315^d, A^	0.06 ± 0.149^d, A^	0.17 ± 0.419^d, A^	0.587

Same lowercase superscript letters in a column represent a statistical difference determined by Mann-Whitney test and Kruskal-Wallis test (*P* < 0.05)

Different uppercase superscript letters in a row represent a statistical difference determined by Kruskal-Wallis test (*P* < 0.05)

*P value calculated among the coronal, middle and apical 1/3 within each group

### Voids distribution along root canal parts in SOI and SCO groups

**Fig. 3:**
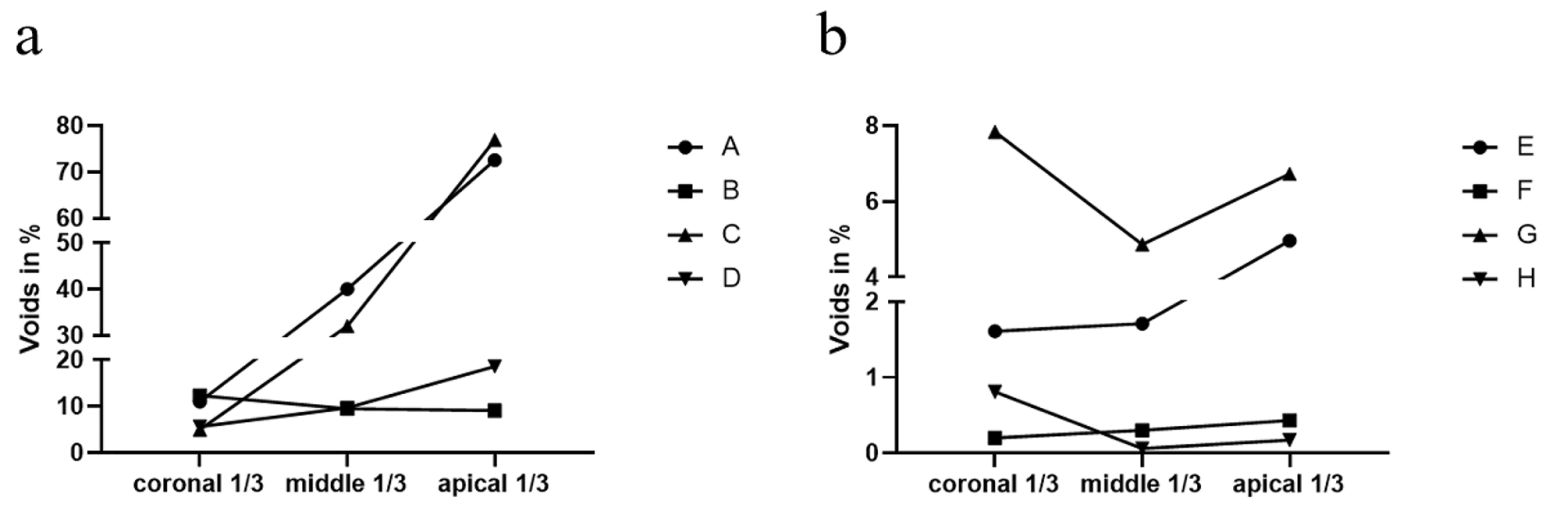
Changes of volumetric percentages of total voids over different root canal parts of each group. (**a**) Volumetric percentage of voids from the coronal to apical parts in SOI groups (A ∼ D); (**b**) Volumetric percentage of voids from the coronal to apical parts in SCO groups (E ∼ H).

For the volumetric percentage of voids in different root canal parts, there was a significant increasing trend from the coronal to apical parts in groups A, C and E (Table 1; Fig. 3). No significant differences were found in the remaining five groups.

### Differences of closed and open voids in SCO groups

Table 2 further demonstrated the differences between closed and open voids in SCO groups (groups E ∼ H). Almost all voids were open voids despite groups or root canal parts. However, the commercial needle groups (E and G) produced significantly more open voids than closed voids throughout the different root canal parts (*P* < 0.05), while no significant differences were observed between the two types of voids in modified needle groups (F and H) throughout different root canal parts (*P* > 0.05). The modified needle groups produced significantly less open voids throughout different root canal parts than the commercial needle groups despite the root canal taper (E vs. F, G vs. H, *P* < 0.05). No obvious differences were found in the volumetric percentages of open voids between different canal tapers using the same kind of needle (E vs. G, F vs. H, *P* > 0.05). Furthermore, no significant differences were observed in volumetric percentages of closed voids among all groups (E vs. F, G vs. H, E vs. G, F vs. H, *P* > 0.05). For the distribution of open voids at different root canal parts within each group, only group E exhibited significantly increased open voids in the apical 1/3 (*P* < 0.05). As to the closed voids, there was no significant difference at different root canal parts within all four groups (*P* > 0.05).

**Table 2:** Volumetric Percentages of Closed and Open Voids (mean ± SD) in SCO Groups

Group	Overall Volume of Voids (%)		Volume of Voids in Coronal 1/3 (%)		Volume of Voids in Middle 1/3 (%)		Volume of Voids in Apical 1/3 (%)		*P* value*	
	Closed	Open	Closed	Open	Closed	Open	Closed	Open	Closed	Open
E	0.00 ± 0.000^A^	2.04 ± 0.761^a, A^	0.00 ± 0.000^B^	1.61 ± 0.596^a, B^	0.00 ± 0.000^C^	1.71 ± 2.116^a, C^	0.00 ± 0.000^D^	4.96 ± 2.163^a, D^	1.000	0.019
F	0.00 ± 0.000	0.26 ± 0.302^a^	0.00 ± 0.000	0.20 ± 0.311^a^	0.00 ± 0.000	0.30 ± 0.685^a^	0.00 ± 0.000	0.43 ± 0.557^a^	1.000	0.822
G	0.12 ± 0.196^A^	4.74 ± 2.958^b, A^	0.32 ± 0.581^B^	7.52 ± 8.478^b, B^	0.00 ± 0.000^C^	4.86 ± 3.920^b, C^	0.00 ± 0.000^D^	6.53 ± 4.399^b, D^	0.120	0.810
H	0.00 ± 0.000	0.46 ± 0.767^b^	0.00 ± 0.000	0.81 ± 1.315^b^	0.00 ± 0.000	0.06 ± 0.149^b^	0.00 ± 0.000	0.17 ± 0.419^b^	1.000	0.587

Same lowercase superscript letters in a column represent a statistical difference determined by Mann-Whitney test (*P* < 0.05)

Same uppercase superscript letters in a row represent a statistical difference determined by Mann-Whitney test (*P* < 0.05)

*P value calculated using Kruskal-Wallis test for closed and open voids respectively among the coronal, middle and apical 1/3 within each group

## Discussion

The quality of root canal obturation is an important factor affecting the prognosis of root canal treatment, as unsatisfactory obturation could lead to root canal reinfection and periapical diseases [19]. It is reported that flawed root canal filling could account for up to 58% of failed root canal treatments [20]. Intra-canal voids are a major flaw of the unsatisfactory obturation as it would seriously compromise the sealing quality of obturation, especially the voids in apical root canal region. Apical fillings without voids can significantly improve the prognosis of root canal treatment [21]. Various methods and devices have been employed to reduce voids in root canal fillings. Ultrasonic working tips can be used to break vapor lock in the apical region through cavitation and sonophoresis effects [22, 23], but the efficiency for removing voids is quite limited in the isthmus and apical root canal [24, 25]. Studies reported an 88.9%∼93.7% volumetric percentage of fillings in the apical root canal after using ultrasonic vibration with single-cone obturation [25], which seems much lower than the 99.57%∼99.83% of modified needle groups in this study. As for commercial sealer injection needles, such as 27G needle or 30G Navi needles, they are either too thick to reach the apical area of root canals or too thin for the sealer to flow through. Recently, negative pressure systems have been experimentally applied for intra-canal sealer induction, and this method can effectively reduce the apical voids and improve the quality of obturation, which reported a similar overall volumetric percentage of voids (0.33%) as that observed in this study (0.26%∼0.46%) [16]. Despite this, a special negative pressure device is needed and the parameter needs to be carefully adjusted for optimal results [16, 26]. The modified deflation sealer injection needle used in this study can be made using handy materials and would be easy to use in clinical situations.

In our previous study [18], the modified needle had been shown to be able to effectively reduce the voids in both resin and real root canals during the sealer injection. However, in the single-cone obturation technique, a gutta-percha point is required to insert into the canal after the sealer injection. Therefore, to clarify whether the modified needle could also reduce the voids after the insertion of the gutta-percha point, this study was designed to supplement our previous study.

In this study, regardless of the needle type used for sealer injection, intra-canal voids could not be completely eliminated. This result is consistent with previous studies [27, 28]. Despite this, this study demonstrated that modified needles could significantly reduce the intra-canal voids, especially in the apical part of root canal, than commercial needles. In SOI groups, modified needles significantly reduced the voids in the whole root canal, while more voids were found in the apical part of root canal when using the commercial needles despite the canal taper. This may be due to the fact that apical vapor lock effect was evident when the sealer was injected using commercial needles. On the other hand, when using the modified deflation needle, the air in apical area could be passively deflated so that the sealer could enter the apical part of root canal.

In SCO groups, the overall volumetric percentages of voids in groups using commercial needles were consistent with the results of previous studies [6–10]. In all groups, the modified needle effectively improved the obturation quality, especially in the apical 1/3. And in 0.04 tapered groups, the modified needle was proved to be very effective in reducing voids in all root canal parts, which may suggest it is suitable for sealer injection in canals with small tapers. Furthermore, for voids distribution in root canals of each group, the 0.06 tapered group using commercial needles showed more voids in the apical 1/3, while the 0.04 tapered group showed no significant differences in voids distribution along the root. This was probably due to the re-distribution of apical voids toward the middle and coronal canal parts after the inserting of gutta-percha point [16]. In the canal with a large taper, the distance of void moving towards middle and coronal parts would be less than in the canal with small taper. Meanwhile, no significant differences were observed in the distribution of voids for groups using modified needles, which was consistent with the results of SOI groups. In modified needle groups with either 0.06 or 0.04 taper, the insertion of gutta-percha point would not produce significant difference in the re-distribution of voids. Voids in root canal filling material could be classified as closed and open voids based on their locations. Closed voids refer to those voids inside the filling material (sealer and gutta-percha), which are considered to have no significant impact on the prognosis of root canal treatment due to the minimal possibility for these voids to become the pathway or shelter for bacteria invasion [29]. Open voids, however, are connected to the root canal wall and could become the pathway for micro-leakage and bacteria invasion, thus posing high risk for the long-term result of root canal treatment [30]. In this study, almost all voids were open voids in either commercial or modified needle group after the single-cone obturation, which was consistent with the results of previous studies [31, 32]. More importantly, the modified needle can effectively reduce the volumetric percentage of open voids throughout different root canal parts when compared to the commercial needle. The reduced open voids throughout different root canal parts in modified needle groups may mean the improved quality of single-cone obturation. As the open voids might serve as pathways for bacteria to enter root canals, reducing open voids would possibly lower the chance of root canal reinfection.

This study utilized mandibular incisors with single root canal due to their thin straight canal space so that the aimed root canal shape could be well reflected [33]. Moreover, the round shape of the prepared root canal would be suitable for single-cone obturation [34]. As the apical foramen is surrounded by periodontal ligament and alveolar bone in vivo, paraffin was used to seal the apex in this study, simulating the in-vivo conditions. Besides, during the single-cone obturation process in this study, the gutta-percha point was slowly inserted into the sealer-filled root canal to ensure minimal sealers could be pushed out of the apical foramen, which is same as required in the clinical operations.

In previous studies, chemical and microscopic methods such as dye penetration testing, fluid transport method, and scanning electron microscopy analysis are commonly used for evaluating the quality of root canal obturation [35–37]. However, these methods may cause material loss during sample processing, thus affecting the accuracy of analysis [38]. In recent decades, micro-CT has been applied in related studies due to its properties of rapidity, high-precision, and non-destruction to samples [17]. Micro-CT with high resolution can distinguish dentin, filling materials and voids based on the grayscale values, and generate two-dimensional (2D) tomographic images through scanning. These 2D images could be transformed into 3D reconstructions that accurately display the stereoscopic morphology and spatial relationships of dentin, filling material and voids [10, 39–42], which enables quantitative and qualitative assessments of the quality of root canal obturation [43]. The resolution of micro-CT plays a crucial role in analyzing voids, affecting the accuracy of results [44]. Therefore, in this study, a high resolution (6 μm) was used to obtain accurate images for voids analysis.

Despite the findings of this study, due to the small sample size, the limited type of root canal, and the lack of in vivo studies, the efficiency and stability of this modified passive deflation sealer injection needle still needs further investigations.

## Conclusions

Based on the findings of this study, the modified passive deflation sealer injection needle could effectively improve the quality of single-cone obturation through reducing intra-canal voids, especially open voids throughout the root canal, thus might possibly be developed to an effective intra-canal sealer delivering instrument. Despite this, more in vitro and in vivo studies need be conducted to further test the efficiency and stability of this modified needle.

## Data Availability

All data generated or analyzed during this study are included in this published article.

## Abbreviations

Micro-CT: Micro-computed tomography
SOI: Sealer-only injection
SCO: Single-cone obturation with sealer and gutta-percha
2D: Two dimension
3D: Three dimension
SD: Standard deviation
ANOVA: Analysis of variance
